# Ethnic differences in the association between age at diagnosis of diabetes and the risk of cardiovascular complications: a population-based cohort study

**DOI:** 10.1186/s12933-023-01951-z

**Published:** 2023-09-04

**Authors:** Calvin Ke, Thérèse A. Stukel, Deva Thiruchelvam, Baiju R. Shah

**Affiliations:** 1 Department of Medicine, University of Toronto, Toronto, ON Canada; 2 Department of Medicine, Toronto General Hospital, University Health Network, 12 E-252, 200 Elizabeth St, Toronto, ON M5G 2C4 Canada; 3 ICES, Toronto, ON Canada; 4 Institute of Health Policy, Management, and Evaluation, University of Toronto, Toronto, ON Canada; 5 Department of Medicine, Sunnybrook Hospital, Toronto, ON Canada

**Keywords:** Diabetes mellitus, Type 2, Age of onset, Ethnicity, Cardiovascular diseases, Epidemiology, Health status disparities

## Abstract

**Background:**

We examined ethnic differences in the association between age at diagnosis of diabetes and the risk of cardiovascular complications.

**Methods:**

We conducted a population-based cohort study in Ontario, Canada among individuals with diabetes and matched individuals without diabetes (2002-18). We fit Cox proportional hazards models to determine the associations of age at diagnosis and ethnicity (Chinese, South Asian, general population) with cardiovascular complications. We tested for an interaction between age at diagnosis and ethnicity.

**Results:**

There were 453,433 individuals with diabetes (49.7% women) and 453,433 matches. There was a significant interaction between age at diagnosis and ethnicity (*P* < 0.0001). Young-onset diabetes (age at diagnosis < 40) was associated with higher cardiovascular risk [hazard ratios: Chinese 4.25 (3.05–5.91), South Asian: 3.82 (3.19–4.57), General: 3.46 (3.26–3.66)] than usual-onset diabetes [age at diagnosis ≥ 40 years; Chinese: 2.22 (2.04–2.66), South Asian: 2.43 (2.22–2.66), General: 1.83 (1.81–1.86)] versus ethnicity-matched individuals. Among those with young-onset diabetes, Chinese ethnicity was associated with lower overall cardiovascular [0.44 (0.32–0.61)] but similar stroke risks versus the general population; while South Asian ethnicity was associated with lower overall cardiovascular [0.75 (0.64–0.89)] but similar coronary artery disease risks versus the general population. In usual-onset diabetes, Chinese ethnicity was associated with lower cardiovascular risk [0.44 (0.42–0.46)], while South Asian ethnicity was associated with lower cardiovascular [0.90 (0.86–0.95)] and higher coronary artery disease [1.08 (1.01–1.15)] risks versus the general population.

**Conclusions:**

There are important ethnic differences in the association between age at diagnosis and risk of cardiovascular complications.

**Supplementary Information:**

The online version contains supplementary material available at 10.1186/s12933-023-01951-z.

## Background

Young-onset diabetes (defined here as age at diagnosis < 40 years) is associated with greater cardiovascular risk compared with usual-onset diabetes (age at diagnosis ≥ 40 years) [1]. In a registry-based analysis of the Swedish population, people with young-onset diabetes had more than triple the risk of developing cardiovascular disease than those with usual-onset diabetes compared to age-matched individuals without diabetes [hazard ratios: young-onset, 3.47 (3.09–3.90); usual-onset, (age at diagnosis 51–60) 1.69 (1.64–1.74)] [2]. Similar associations with cardiovascular and other complications have been independently reported in the US [3], Hong Kong [4], Australia [5–7], and other populations [1, 8, 9]. However, it is unclear whether the association between young-onset diabetes and cardiovascular risk varies according to ethnicity, as meta-analyses and systematic comparisons across these populations have been limited by substantial heterogeneity (*I*^*2*^ ≥ 48%) [1] likely stemming from differences in health care systems across countries.

Phenotypic differences in diabetes across ethnicities might influence cardiovascular risk differently across ages. For example, Chinese and South Asian populations with diabetes have younger ages at diagnosis, and greater tendencies toward insulin deficiency and low or normal body mass index than European populations with diabetes [10]. We conducted a population-based cohort study in Ontario, Canada to examine ethnic differences (Chinese, South Asian, general population) in the association between age at diagnosis and the risk of cardiovascular complications among individuals with diabetes compared to matched individuals without diabetes. We hypothesized that there is a significant interaction between ethnicity and age at diagnosis.

## Methods

### Study design

This was a population-based retrospective matched cohort study in Ontario, Canada using administrative health data from April 1, 2002 to March 31, 2018.

### Setting and data sources

Ontario is Canada’s most populous province. The Ontario Health Insurance Plan (OHIP) funds physician and hospital services for all Ontario residents. We accessed physician billing codes and hospital discharge abstracts using the OHIP database and Canadian Institute for Health Information Discharge Abstract Database respectively. The Ontario Diabetes Database includes people with diabetes identified using a validated algorithm based on physician billing codes and discharge abstracts (positive predictive value [PPV] 91.4%, sensitivity 79.9%; > 90–95% of adults have type 2 diabetes) [11, 12]. We used the Registered Persons Database to identify individuals without diabetes. Because self-identified ethnicity was not available, we defined South Asian and Chinese ethnicity using a validated surname algorithm (PPV 89.3%, 91.9% respectively) [13]. We classified the remainder as the “general population,” which consists of White people (> 80%) and various other ethnicities [14]. We defined the income quintile based on the median household income of each individual’s neighbourhood of residence using the chronologically closest census date. These datasets were linked using unique encoded identifiers and analyzed at ICES.

### Study population

We included all adult (aged ≥ 20 years) residents of Ontario with incident diabetes diagnosed between April 1, 2002 to March 31, 2012. We matched each individual with diabetes to another individual without diabetes, by ethnicity, sex, and year of birth (± 1 year). We excluded all individuals with pre-existing cardiovascular disease. We limited the study to residents of Ontario for at least 2 years before the index date to avoid including recent immigrants with pre-existing diabetes. Because most Chinese and South Asian people live in large urban centres, we excluded all rural residents.

### Exposure and outcomes

The primary exposures were ethnicity and age at diagnosis. The primary composite outcome was hospitalization for coronary artery disease (defined as acute myocardial infarction, percutaneous catheter intervention, or coronary artery bypass graft surgery), stroke, congestive heart failure, peripheral revascularization, or non-traumatic and non-malignant lower extremity amputation (Additional file 1: Table S1). The secondary outcomes included all-cause mortality and each component of the primary outcome. We followed patients until March 31, 2018, censoring upon departure from Ontario.

### Statistical analysis

We described the baseline characteristics and computed standardized differences [15]. We fit Cox proportional hazards models using attained age as the timescale. Individuals with diabetes entered the study together with their match at the date of diabetes diagnosis. We computed adjusted hazard ratios (HRs) with 95% confidence intervals CI to determine the associations of age at diagnosis and ethnicity with cardiovascular complications, adjusting for pre-specified covariates (sex, ethnicity, income quintile; Additional file 1: Figure S1). We accounted for matched sets as clusters, and obtained robust sandwich estimates of the variance [16].

We tested for an interaction between age at diagnosis and ethnicity. As this interaction was significant (*P* < 0.0001), we included an interaction term between age at diagnosis and ethnicity in all models. We characterized this interaction by expressing the HRs using 2 alternate reference populations. Firstly, to examine the variation in cardiovascular risk among people with and without diabetes *within* each ethnicity, we compared individuals with diabetes to matched individuals without diabetes as the reference population, separately for each ethnicity. Secondly, to examine the variation in cardiovascular risk among people with diabetes *across* ethnicities, we compared Chinese and South Asian individuals with diabetes to individuals in the general population with diabetes as the reference group. We verified the proportionality assumption using Schoenfeld residuals.

To assess the effect of death as a competing risk, we conducted a sensitivity analysis using a Fine and Gray subdistribution hazard competing risk model. To assess for secular effects, we conducted a sensitivity analysis adjusting for calendar year. Missing socioeconomic status data were minimal (< 0.2%) and handled by complete cases analysis. We used SAS Enterprise Guide version 7.1 (Cary, NC) for all analyses. The study was approved by the University of Toronto Research Ethics Board.

## Results

Baseline characteristics of the study population are shown in Table 1. There were 453,433 individuals with diabetes (young-onset *n* = 47,710, usual-onset *n* = 405,723; 49.7% women), including 15,977 (3.5%) South Asian and 20,321 (4.5%) Chinese people. The median follow-up time was 9.6 years. In the general population, the socioeconomic status distribution was lower among people with diabetes than the matched population. Previous comorbidities were uncommon.

**Table 1:** Baseline characteristics of the study cohort, captured from 1 April 2002 to 31 March 2012, stratified by age of diabetes diagnosis

	Young-onset diabetes	Matches	Standardized difference	Usual-onset diabetes	Matches	Standardized difference
	N = 47,710	N = 47,710		N = 405,723	N = 405,723	
Age (years; mean ± standard deviation)	33.3 ± 4.9	33.3 ± 4.9	0.00	59.8 ± 11.8	59.8 ± 11.8	0.00
Women	24,939 (52.3%)	24,939 (52.3%)	0.00	200,205 (49.3%)	200,205 (49.3%)	0.00
Ethnicity						
South Asian	4,084 (8.6%)	4084 (8.6%)	0.00	11,893 (2.9%)	11,893 (2.9%)	0.00
Chinese	1,716 (3.6%)	1716 (3.6%)	0.00	18,605 (4.6%)	18,605 (4.6%)	0.00
General	41,910 (87.8%)	41,910 (87.8%)	0.00	375,225 (92.5%)	375,225 (92.5%)	0.00
Socioeconomic status quintile						
1 (lowest)	13,342 (28.0%)	10,105 (21.2%)	0.16	89,331 (22.0%)	69,980 (17.2%)	0.12
2	10,725 (22.5%)	9939 (20.8%)	0.04	88,940 (21.9%)	77,530 (19.1%)	0.07
3	9657 (20.2%)	9478 (19.9%)	0.01	82,202 (20.3%)	78,884 (19.4%)	0.02
4	8279 (17.4%)	9620 (20.2%)	0.07	77,265 (19.0%)	84,646 (20.9%)	0.05
5 (highest)	5575 (11.7%)	8490 (17.8%)	0.17	67,277 (16.6%)	94,042 (23.2%)	0.17
Primary care visits (median, IQR)	4 (2–8)	2 (0–4)	0.65	5 (2–8)	3 (1–6)	0.50

Values are counts and percentages unless otherwise indicated. A standardized difference of ≥ 0.1 is considered significant [15]

*IQR* interquartile range

**Fig. 1:**
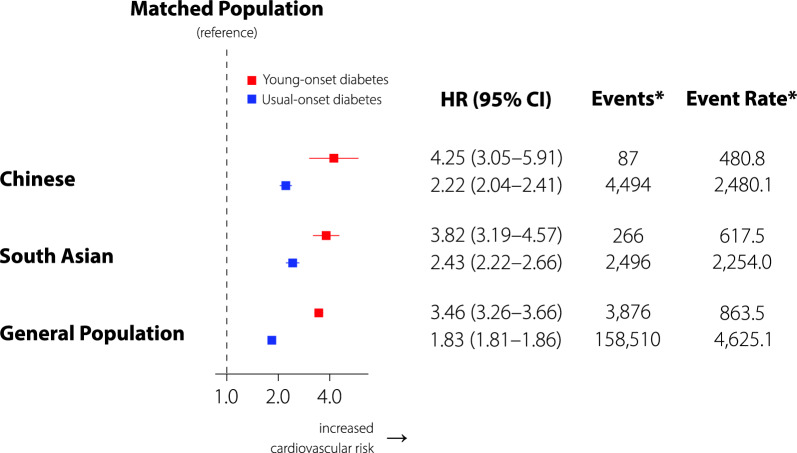
Interaction between ethnicity and age at diagnosis of diabetes (*P* < 0.0001 for interaction) in the risk of cardiovascular events among adults in Ontario, Canada. Hazard ratios of cardiovascular events (coronary artery disease, congestive heart failure, stroke, peripheral revascularization, and lower extremity amputation) are shown for young- and usual-onset diabetes compared to matches without diabetes, stratified by ethnicity (Chinese, South Asian, general population). See Additional file 1: Figure. S2 for secondary outcomes. *Events and event rates pertain to the cases. Event rates are per 100,000 person-years

Figure 1 shows the HRs for individuals with diabetes versus matches of shared ethnicity, stratified by age at diagnosis. Young-onset diabetes was consistently associated with a 3.5- to fourfold elevation in the risk of cardiovascular complications versus the matched population [HR: Chinese 4.25 (3.05–5.91), South Asian: 3.82 (3.19–4.57), General: 3.46 (3.26–3.66)], while usual-onset diabetes was consistently associated with around double the risk of cardiovascular complications versus the matched population [Chinese: 2.22 (2.04–2.41), South Asian: 2.43 (2.22–2.66), General: 1.83 (1.81–1.86)]. Results for the secondary outcomes showed broadly similar patterns except for stroke in South Asian participants, where the HRs were similar for young- and usual-onset diabetes (Additional file 1: Figure S2). Peripheral revascularization and amputation were rare (Additional file 1: Table S2).

**Fig. 2:**
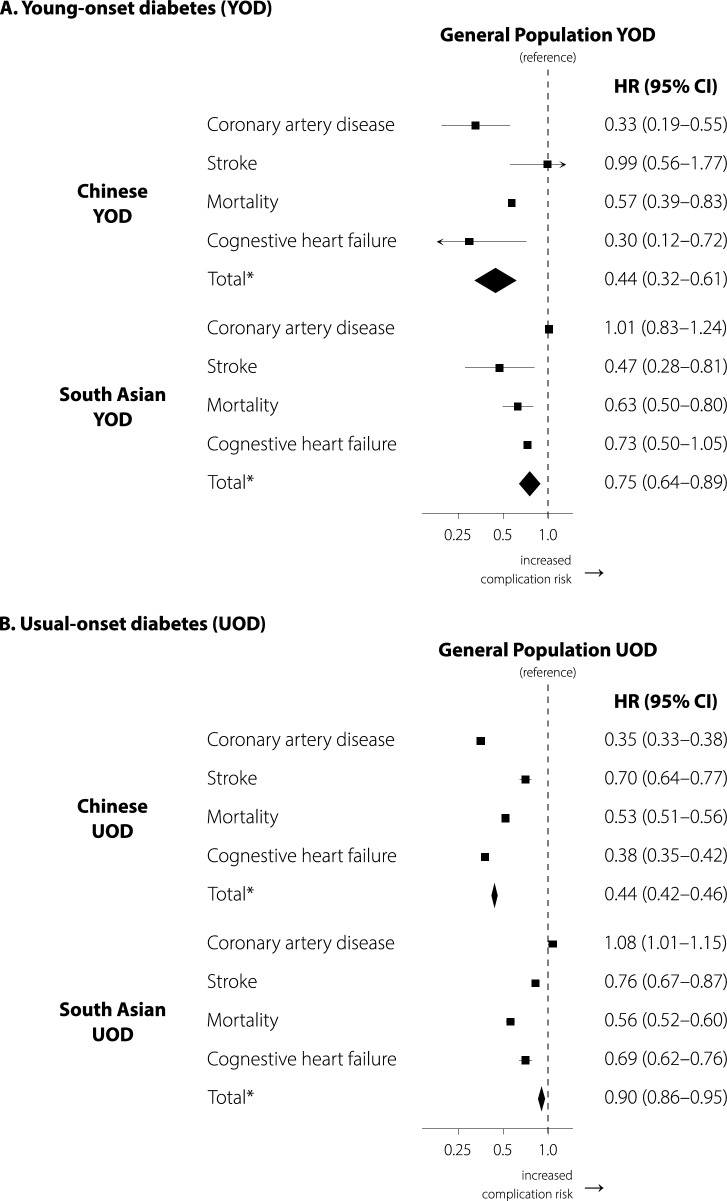
Interaction between ethnicity and age at diagnosis of diabetes (*P* < 0.0001 for interaction) in the risk of cardiovascular events among adults in Ontario, Canada. Hazard ratios are shown for Chinese and South Asian people compared with the general population (reference) for (**A**) young-onset diabetes and (**B**) usual-onset diabetes. *includes coronary artery disease, congestive heart failure, stroke, peripheral revascularization, or non-traumatic and non-malignant lower extremity amputation. Amputation and peripheral revascularization were rare (see Additional file 1: Table S2 for these secondary outcomes)

Figure 2 shows the HRs comparing the Chinese, South Asian, and general populations with diabetes, stratified by age at diagnosis. The following results pertain to people with young-onset diabetes. Chinese ethnicity was associated with a similar hazard of stroke [0.99 (0.56–1.77)], lower hazards of other complications aside from stroke, and a 56% lower hazard of overall complications compared to the general population [0.44 (0.32–0.61)]. By contrast, South Asian ethnicity was associated with a similar hazard of coronary artery disease [1.01 (0.83–1.24)], lower hazards of other complications aside from coronary artery disease, and a 25% lower hazard of overall complications compared to the general population [0.75 (0.64–0.89)].

**Fig. 3:**
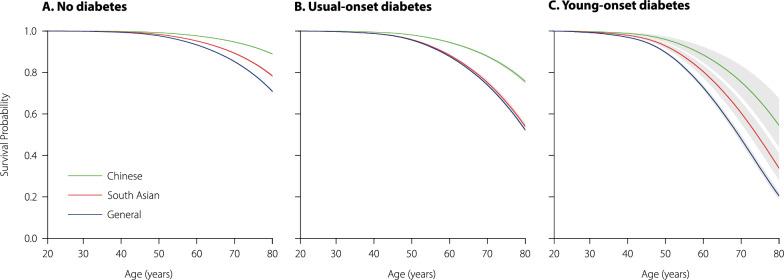
Kaplan–Meier survival curves for cardiovascular events in individuals without diabetes (**A**), usual-onset diabetes (**B**), and young-onset diabetes (**C**), stratified by ethnicity, shown for ages 20 to 80 years. Cox proportional hazards models were fit using age as the timescale. The shaded areas indicate 95% confidence intervals (some confidence intervals are too small to be visible)

Among people with usual-onset diabetes, Chinese ethnicity was associated with 56% lower hazard of cardiovascular complications compared to the general population [0.44 (0.42–0.46)]; results for the secondary outcomes were consistent in direction. However, South Asian ethnicity was associated with an 8% higher hazard of coronary artery disease [1.08 (1.01–1.15)], lower hazards of other complications, and a 10% lower hazard of overall complications compared to the general population [0.90 (0.86–0.95)]. The corresponding Kaplan–Meier survival curves are shown in Fig. 3. Results of the sensitivity analyses were consistent with the main results (Additional file 1: Tables S3, S4).

## Conclusions

This large multi-ethnic, population-based study is the first to our knowledge to show that although young-onset diabetes is consistently associated with around four-fold higher cardiovascular risk compared to diabetes-free individuals of shared ethnicity, Chinese and South Asian people with young-onset diabetes have substantially different complication risks relative to the general population. Chinese people diagnosed with diabetes at any age have a lower overall cardiovascular risk compared to the general population, yet Chinese people with young-onset diabetes have an especially high risk of stroke that is similar in magnitude to the general population. South Asian individuals with young-onset diabetes have a lower overall cardiovascular risk, but a similarly high risk of coronary artery disease, compared to the general population. By contrast, South Asian individuals with usual-onset diabetes have a lower overall cardiovascular risk and a slightly higher coronary artery disease risk versus the general population. These outcome disparities may reflect the heterogeneous phenotypes of diabetes in Chinese and South Asian populations, and suggest that paradigms of cardiovascular risk prediction and management should be carefully reconsidered to account for the important interaction between ethnicity and age at diagnosis.

Our findings revealed that Chinese people with young-onset diabetes—but not usual-onset diabetes—have as high a risk of stroke as the general population with young-onset diabetes. These results extend previous Canadian studies, which did not account for age at diagnosis, and reported that Chinese adults with diabetes have a similar risk of stroke and lower risks of other cardiovascular complications compared to the general population [17, 18]. By contrast, the international Action in Diabetes and Vascular Disease: Preterax and Diamicron Modified Release Controlled Evaluation (ADVANCE) study, where “Asian” (predominantly Chinese) adults with diabetes had double the risk of stroke (HR 2.02, 1.62–2.51) and a lower risk of coronary artery disease (HR 0.72, 0.60–0.85) compared with Europeans [19]. However, the ADVANCE study did not account for the substantial differences in clinical practice and health systems across countries. Our findings are consistent with previous studies conducted in China, including a national cross-sectional study reporting that people with young-onset diabetes had a higher odds of non-fatal cardiovascular disease [odds ratio 1.91 (1.81–2.02)] [20]. In the Hong Kong Diabetes Registry, people with young-onset diabetes had a higher hazard of cardiovascular complications versus people with usual-onset diabetes [HR 1.48 (1.17–1.88)] [4]. Taken together, this evidence suggests that relative to the general population, Chinese people with diabetes might benefit from a greater focus on stroke prevention [21]—especially for young-onset diabetes.

Conversely, we found that disparities in coronary artery disease between the South Asian and general populations worsened with increasing age at diagnosis of diabetes. Previous studies in Canada and the UK reported that South Asian individuals with diabetes have similar risks of coronary artery disease and overall cardiovascular complications as the general population, but did not account for age at diagnosis [17, 18, 22]. Our study reveals that young-onset diabetes in South Asian individuals is associated with similar coronary artery disease risk, but otherwise relatively lower cardiovascular risk than the general population. This benefit appears to be lost in South Asian individuals with usual-onset diabetes, who had the highest risk of coronary artery disease across ethnicities. Such outcome disparities might arise from systemic inequities due to factors such as structural racism, although previous research suggests that South Asian individuals receive an equitable quality of diabetes care [23]. Alternatively, these variations might reflect distinctive phenotypes or “subtypes” of diabetes, which have been previously classified based on age at diagnosis and other characteristics. In the “WellGen” cohort of Indians aged 20–45 years at diabetes diagnosis, over half had “severe insulin-deficient diabetes,” which was associated with a much lower prevalence of cardiovascular disease compared to “mild age-related diabetes” (coronary artery disease: 6.0%, 12.6% respectively; stroke 1.6%, 3.1% respectively) [24]. These results require further confirmation, as small differences in attained age were not accounted for [25, 26]. Our results suggest that better strategies to prevent coronary artery disease may be useful in the South Asian population, especially those with usual-onset diabetes.

This interaction between age at diagnosis and ethnicity carries important implications for personalizing cardiovascular risk prediction and management. Our results extend the findings of previous studies showing that cardiovascular risk prediction algorithms developed in populations of European origin provide inaccurate estimates when applied to Chinese and South Asian populations [27, 28]. Studies classifying diabetes into subtypes of potential prognostic importance have suggested that unlike European populations, young-onset diabetes is especially driven by insulin deficiency in both Chinese and South Asian populations [10]. Insulin-deficient diabetes might be associated with lower risk of cardiovascular disease compared to insulin-resistant diabetes driven by obesity [25, 29]. However, such classifications require measures such as C-peptide and indicators of insulin secretion and resistance which are not routinely available in clinical practice, and have failed to identify consistent differences in cardiovascular complications after adjusting for demographic characteristics [25, 26]. We have shown how age at diagnosis and ethnicity are simple, easily ascertained features that have important prognostic value and may inform the individualization of diabetes management and cardiovascular risk reduction [30, 31]﻿. For example, GLP-1 receptor agonists may be more efficacious than SGLT2 inhibitors in preventing stroke [32, 33], appear to be efficacious in youth with young-onset diabetes [34], and may be more efficacious in Chinese and other Asian populations relative to European populations [35]. More research is required to determine the best strategies to lower cardiovascular risk in young-onset diabetes [36, 37],﻿ which was the group with the highest cardiovascular risk at any attained age within each ethnicity.

Strengths of our study include its multiethnic population-based approach that allowed for comparisons across ethnicities and ages at diagnosis with virtually no loss to follow-up. Unlike multi-country comparisons, our study population was contained within a single health system with universal health coverage, thus eliminating confounding due to differences across health systems. Our use of attained age as the timescale allowed us to generate estimates that were robust to extensive sensitivity analyses. However, there are some limitations to note. Chinese and South Asian subpopulations, and other ethnic groups represented in the general population, might exhibit heterogeneous outcomes; further research is required to explore these possibilities [38–40]. We were unable to identify type 1 diabetes and undiagnosed diabetes. However, our findings were likely driven by type 2 diabetes, which represented around 95% of individuals with diabetes [12]. The lower incidence of type 1 diabetes in Chinese and South Asian relative to White people (> 80% of the general population) would bias the results conservatively [29, 41, 42]. We cannot exclude residual confounding due to factors such as blood pressure, lipids, obesity, smoking, and alcohol use.

In conclusion, our study demonstrates how age at diagnosis and ethnicity interact and contribute to the heterogeneity of cardiovascular outcomes among people with diabetes. This interaction yields valuable information to individualize cardiovascular risk prediction and management. The prognostic differences we identified likely reflect how young- and usual-onset diabetes manifest differently across ethnicities, and suggest that young-onset diabetes in Chinese and South Asian individuals, which is primarily driven by insulin deficiency [10], may result in lower risks of some—but not all—cardiovascular complications compared to the general population. Further efforts are required to prevent cardiovascular complications in young-onset diabetes, which was associated with the highest cardiovascular risk within every ethnicity.

### Supplementary Information

**Additional file 1: Table S1. **Outcome definitions used in the study. **Table S2. **Hazard ratios of peripheral revascularization and lower extremity amputation for (1) Chinese and South Asian adults compared with the general population (reference), stratified by age at diagnosis category; and (2) young-onset diabetes (YOD) and usual-onset diabetes (UOD) compared with matches without diabetes (reference), stratified by ethnicity. **Table S3. **Sensitivity analysis using Fine and Gray subdistribution hazard competing risk model. **Table S4. **Sensitivity analysis including an adjustment term for calendar year. **Figure S1. **Causal diagram depicting the clinically significant relationships among age at diagnosis (exposure), cardiovascular complications (outcome), and other variables. **Figure S2. **Hazard ratios of cardiovascular events among adults with YOD and UOD versus matches without diabetes in Ontario, Canada, stratified by ethnicity.

## Data Availability

The dataset from this study is held securely in coded form at ICES. While legal data sharing agreements between ICES and data providers (e.g., healthcare organizations and government) prohibit ICES from making the dataset publicly available, access may be granted to those who meet pre-specified criteria for confidential access, available at www.ices.on.ca/DAS (email: das@ices.on.ca).
