# Supplementary material for: Ethnic differences in the association between age at diagnosis of diabetes and the risk of cardiovascular complications: a population-based cohort study
Source: Cardiovasc Diabetol. 2023 Sep 4;22:241. doi: 10.1186/s12933-023-01951-z (PMC10476404; doi:10.1186/s12933-023-01951-z)
Supplement: Supplementary file 1 — Additional file 1: Table S1. Outcome definitions used in the study. Table S2. Hazard ratios of peripheral revascularization and lower extremity amputation for (1) Chinese and South Asian adults compared with the general population (reference), stratified by age at diagnosis category; and (2) young-onset diabetes (YOD) and usual-onset diabetes (UOD) compared with matches without diabetes (reference), stratified by ethnicity. Table S3. Sensitivity analysis using Fine and Gray subdistribution hazard competing risk model. Table S4. Sensitivity analysis including an adjustment term for calendar year. Figure S1. Causal diagram depicting the clinically significant relationships among age at diagnosis (exposure), cardiovascular complications (outcome), and other variables. Figure S2. Hazard ratios of cardiovascular events among adults with YOD and UOD versus matches without diabetes in Ontario, Canada, stratified by ethnicity. [file 12933_2023_1951_MOESM1_ESM.pdf]

Additional File

Table S1 Outcome definitions used in the study

Outcome	Definition
Primary Composite Outcome	• Hospitalization for acute myocardial infarction (ICD-9 410; ICD-10 I21-22 from CIHI-DAD)<br>• Hospitalization for percutaneous coronary intervention (ICD-9 CCP 4802, 4803; ICD-10 CCI 1IJ50, 1IJ54, 1IJ57GQ from CIHI-DAD or CIHI-SDS)<br>• Hospitalization for coronary artery bypass surgery (ICD-9 CCP 481, ICD-10 CCI 1IJ76 from CIHI-DAD)<br>• Hospitalization for congestive heart failure (ICD-9 428; ICD-10 I50)<br>• Hospitalization for stroke (ICD-9 430, 431, 434, 436, 363.2; ICD-10 I60, I61, I63 [excluding I63.6], I64, H34.1 from CIHI-DAD)<br>• Hospitalization for peripheral revascularization procedure (ICD-9 CCP 50.18, 51.25, 51.29; ICD-10 CCI 1KG50, 1KG57, 1KG76, 1KG35HAC1, 1KG35HHC1, 1KA76MZ, 1KE76MZ, 1KT76MZ from CIHI-DAD)<br>• Hospitalization for major lower extremity amputation (below hip and above ankle; ICD-9 CCP 96.14, 96.15; ICD-10 CCI 1VC93, 1VG93, 1VQ93 from CIHI-DAD) or minor lower extremity amputation (ankle or lower; ICD-9 CCP 96.11, 96.12, 96.13; ICD-10 CCI: 1WL93, 1WA93, 1WE93, 1WJ93, 1WM93, 1WI93, 1WK93, 1WN93 from CIHI-DAD)*<br>o *Excluding amputations occurring during a hospitalization related to malignancy or major trauma (defined as ICD-9 170.7, 170.8, 176.1, 171.3, 213.7, 213.8, 215.3, 820-829, 835, 836, 837, 838, 843, 844, 845, 904, 928, 929, 956, 957, 895, 896, 897, 945-946, 991.2, 991.3; ICD10 C40.2, C40.3, C46.1, C47.2, C49.2, D16.2, D16.3, D21.2, S72-S79, S82-S89, S92-S99, T02.3, T02.5-T02.9, T03.3-T03.9, T04.3-T04.9, T05.3-05.9, T07, T12, T13.2-T13.9, T14.2-T14.9, T24-T25, T34.6-T35.1, T35.5-T35.7)
Secondary Outcomes	• All-cause mortality<br>• Hospitalization for coronary artery disease, defined as:<br>o Hospitalization for acute myocardial infarction (ICD-9 410; ICD-10 I21-22 from CIHI-DAD);<br>o Hospitalization for percutaneous coronary intervention (ICD-9 CCP 4802, 4803; ICD-10 CCI 1IJ50, 1IJ54, 1IJ57GQ from CIHI-DAD or CIHI-SDS); or

	o Hospitalization for coronary artery bypass surgery (ICD-9 CCP 481, ICD-10 CCI 1IJ76 from CIHI-DAD)<br>• Hospitalization for congestive heart failure (ICD-9 428; ICD-10 I50)<br>• Hospitalization for stroke (ICD-9 430, 431, 434, 436, 363.2; ICD-10 I60, I61, I63 [excluding I63.6], I64, H34.1 from CIHI-DAD)<br>• Hospitalization for peripheral revascularization procedure (ICD-9 CCP 50.18, 51.25, 51.29; ICD-10 CCI 1KG50, 1KG57, 1KG76, 1KG35HAC1, 1KG35HHC1, 1KA76MZ, 1KE76MZ, 1KT76MZ from CIHI-DAD)<br>• Hospitalization for major lower extremity amputation (below hip and above ankle; ICD-9 CCP 96.14, 96.15; ICD-10 CCI 1VC93, 1VG93, 1VQ93 from CIHI-DAD) or minor lower extremity amputation (ankle or lower; ICD-9 CCP 96.11, 96.12, 96.13; ICD-10 CCI: 1WL93, 1WA93, 1WE93, 1WJ93, 1WM93, 1WI93, 1WK93, 1WN93 from CIHI-DAD)*<br>o *Excluding amputations occurring during a hospitalization related to malignancy or major trauma (defined as ICD-9 170.7, 170.8, 176.1, 171.3, 213.7, 213.8, 215.3, 820-829, 835, 836, 837, 838, 843, 844, 845, 904, 928, 929, 956, 957, 895, 896, 897, 945-946, 991.2, 991.3; ICD10 C40.2, C40.3, C46.1, C47.2, C49.2, D16.2, D16.3, D21.2, S72-S79, S82-S89, S92-S99, T02.3, T02.5-T02.9, T03.3-T03.9, T04.3-T04.9, T05.3-05.9, T07, T12, T13.2-T13.9, T14.2-T14.9, T24-T25, T34.6-T35.1, T35.5-T35.7)

Abbreviations: CCI, Canadian Classification of Health Interventions; CCP, Canadian Classification of Diagnostic, Therapeutic and Surgical Procedures; ICD, International Classification of Diseases

We limited the matched population to those who were never diagnosed with diabetes according to a validated highly-sensitive algorithm (sensitivity 93.6%)[8] through March 31, 2018, which was the maximum follow-up date.

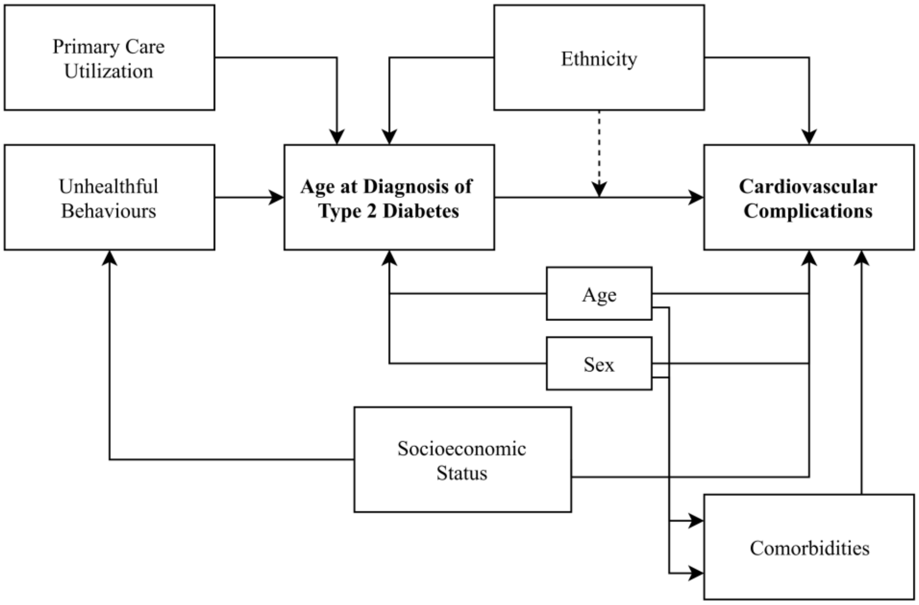

Figure S1 Causal diagram depicting the clinically significant relationships among age at diagnosis (exposure), cardiovascular complications (outcome), and other variables. Under this conceptualization, primary care utilization and comorbidities are not confounding variables.

		Hazard Ratio (95% confidence interval)	Events	Follow-Up*	Event Rate*
**Mortality**					
Chinese	UOD cases vs. controls	1.85 (1.73, 1.99)	2,057	682	1,101.6
	YOD cases vs. controls	3.13 (2.13, 4.60)	27	67	147.9
South Asian	UOD cases vs. controls	1.78 (1.60, 1.98)	862	425	740.7
	YOD cases vs. controls	3.16 (2.47, 4.05)	73	160	167.1
General	UOD cases vs. controls	1.33 (1.31, 1.34)	72,050	13,459	1,955.3
	YOD cases vs. controls	2.10 (1.96, 2.26)	1,158	1,666	253.9
**Coronary artery disease**					
Chinese	UOD cases vs. controls	2.34 (2.03, 2.69)	4,363	672	2,372.7
	YOD cases vs. controls	3.17 (1.85, 5.43)	68	66	374.2
South Asian	UOD cases vs. controls	2.61 (2.31, 2.94)	2,305	409	2,056.7
	YOD cases vs. controls	3.60 (2.89, 4.49)	233	158	539.2
General	UOD cases vs. controls	1.88 (1.85, 1.91)	155,758	12,873	4,419.4
	YOD cases vs. controls	2.76 (2.56, 2.98)	3,181	1,649	704.5
**Congestive heart failure**					
Chinese	UOD cases vs. controls	2.47 (2.13, 2.86)	3,992	677	2,154.6
	YOD cases vs. controls	3.96 (1.63, 9.62)	57	67	312.8
South Asian	UOD cases vs. controls	2.50 (2.09, 2.99)	1,737	421	1,508.2
	YOD cases vs. controls	5.50 (3.71, 8.16)	164	159	376.5
General	UOD cases vs. controls	2.09 (2.05, 2.14)	138,323	13,171	3,835.8
	YOD cases vs. controls	4.34 (3.79, 4.97)	2,531	1,660	556.8
**Stroke**					
Chinese	UOD cases vs. controls	2.13 (1.83, 2.48)	4,160	676	2,248.2
	YOD cases vs. controls	5.49 (3.06, 9.85)	64	66	351.7
South Asian	UOD cases vs. controls	1.85 (1.49, 2.29)	1,766	422	1,527.1
	YOD cases vs. controls	2.10 (1.20, 3.68)	156	159	357.5
General	UOD cases vs. controls	1.56 (1.52, 1.60)	143,516	13,303	3,940.4
	YOD cases vs. controls	2.85 (2.44, 3.31)	2,509	1,662	551.4

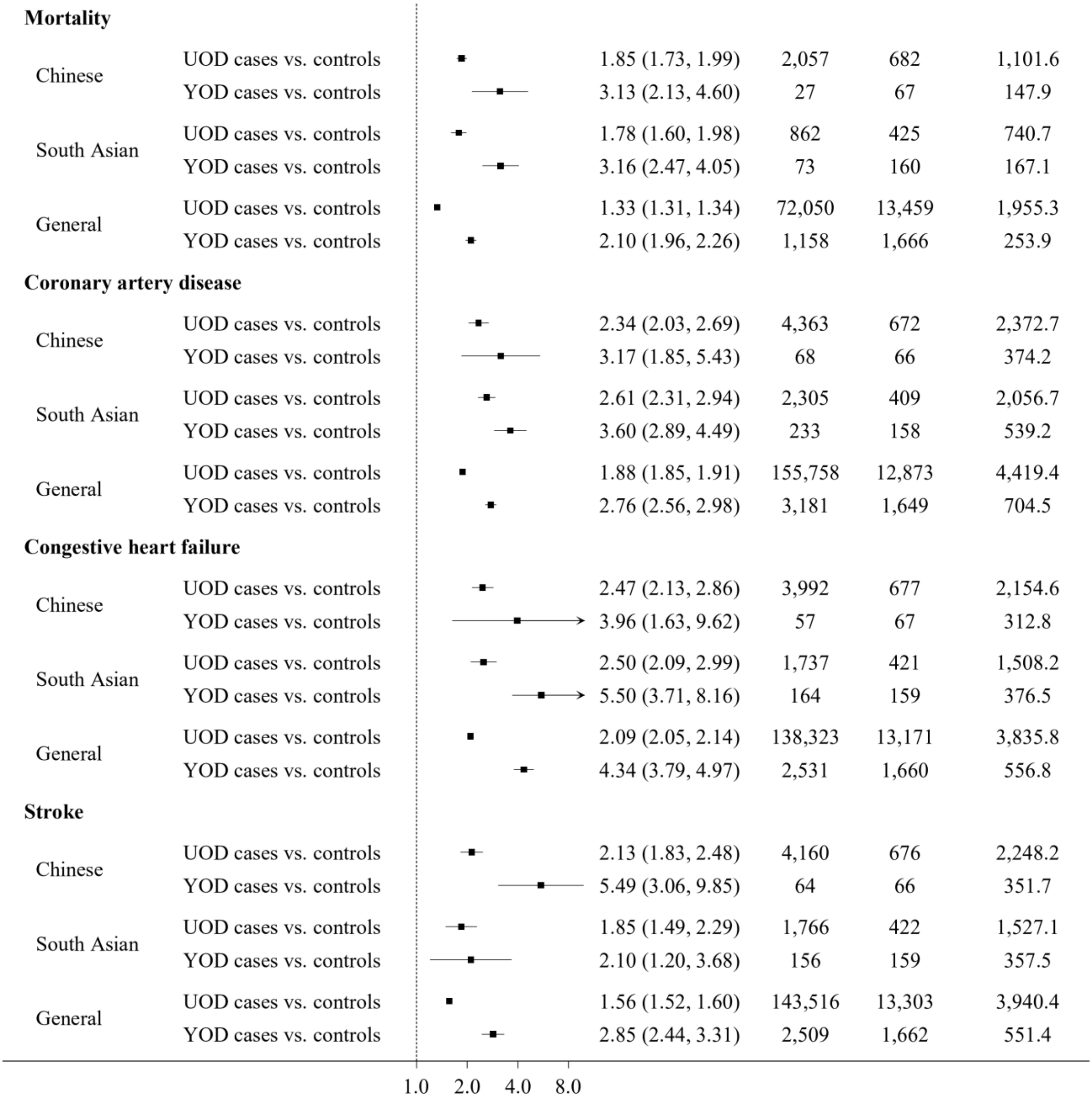

Figure S2 Hazard ratios of cardiovascular events among adults with YOD and UOD versus matches without diabetes in Ontario, Canada, stratified by ethnicity (Chinese, South Asian, general population). For amputation and peripheral revascularization, see Table S2.

*Follow-up times are expressed as 100,000 person-years. Event rates are per 100,000 person-years.

Table S2 Hazard ratios of peripheral revascularization and lower extremity amputation for (1) Chinese and South Asian adults compared with the general population (reference), stratified by age at diagnosis category; and (2) young-onset diabetes (YOD) and usual-onset diabetes (UOD) compared with matches without diabetes (reference), stratified by ethnicity. Some hazard ratios could not be computed due to low event rates (indicated by grey cells).

		Hazard Ratio (95% confidence interval)	Events	Follow-Up*	Event Rate*
Peripheral Revascularization					
YOD	Chinese vs. General		54	66.7	295.9
	South Asian vs. General	0.62 (0.19–2.00)	149	159.5	341.1
UOD	Chinese vs. General	0.19 (0.13–0.28)	4,118	681.6	2,206.8
	South Asian vs. General	0.11 (0.05–0.23)	1,727	424.9	1,484.4
Chinese	UOD cases vs. controls	2.49 (1.23–5.01)	4118	681.6	2,206.8
	YOD cases vs. controls		54	66.7	295.9
South Asian	UOD cases vs. controls	1.46 (0.46–4.60)	1,727	424.9	1,484.4
	YOD cases vs. controls	14.20 (3.32–60.77)	149	159.5	341.1
General	UOD cases vs. controls	2.01 (1.88–2.15)	144,499	13,417.9	3,933.4
	YOD cases vs. controls	3.47 (2.43–4.96)	2347	1,665.2	514.8
Amputation					
YOD	Chinese vs. General		1	66.7	5.5
	South Asian vs. General		5	159.5	11.5
UOD	Chinese vs. General		17	681.8	9.1
	South Asian vs. General		15	424.7	12.9
Chinese	UOD cases vs. controls		17	681.8	9.1
	YOD cases vs. controls		1	66.7	5.5
South Asian	UOD cases vs. controls		15	424.7	12.9
	YOD cases vs. controls		5	159.5	11.5
General	UOD cases vs. controls		2,239	13,429.8	60.9
	YOD cases vs. controls		197	1,663.4	43.3

* Follow-up times are expressed as 100,000 person-years. Event rates are per 100,000 person-years.

Table S3 Sensitivity analysis using Fine and Gray subdistribution hazard competing risk model.* Hazard ratios of cardiovascular events (coronary artery disease, congestive heart failure, stroke, peripheral revascularization, and lower extremity amputation) are shown for (1) Chinese and South Asian adults compared with the general population (reference), stratified by age at diagnosis category; and (2) young-onset diabetes (YOD) and usual-onset diabetes (UOD) compared with matches without diabetes (reference), stratified by ethnicity.

		Hazard Ratio (95% confidence interval)
YOD	Chinese vs. General	0.54 (0.30–0.99)
	South Asian vs. General	1.00 (0.74–1.34)
UOD	Chinese vs. General	0.44 (0.40–0.48)
	South Asian vs. General	0.98 (0.89–1.09)
Control	Chinese vs. General	0.40 (0.35–0.45)
	South Asian vs. General	0.65 (0.55–0.76)
Chinese	UOD cases vs. controls	2.03 (1.72–2.39)
	YOD cases vs. controls	4.58 (2.49–2.43)
South Asian	UOD cases vs. controls	2.79 (2.32–3.36)
	YOD cases vs. controls	5.14 (3.70–7.14)
General	UOD cases vs. controls	1.83 (1.79–1.88)
	YOD cases vs. controls	3.34 (2.97–3.75)

*This analysis was performed in a 25% random subsample due to computational limitations.

Table S4 Sensitivity analysis including an adjustment term for calendar year.* Hazard ratios of cardiovascular events (coronary artery disease, congestive heart failure, stroke, peripheral revascularization, and lower extremity amputation) are shown for Chinese and South Asian adults compared with the general population (reference), stratified by age at diagnosis category; and young-onset diabetes (YOD) and usual-onset diabetes (UOD) compared with controls (reference) without diabetes, stratified by ethnicity (Chinese, South Asian, general population).

		Hazard Ratio (95% confidence interval)
YOD	Chinese vs. General	0.54 (0.30–0.99)
	South Asian vs. General	1.00 (0.74–1.34)
UOD	Chinese vs. General	0.44 (0.40–0.48)
	South Asian vs. General	0.99 (0.89–1.09)
Control	Chinese vs. General	0.40 (0.35–0.45)
	South Asian vs. General	0.65 (0.55–0.76)
Chinese	UOD cases vs. controls	2.03 (1.72–2.39)
	YOD cases vs. controls	4.59 (2.49–8.44)
South Asian	UOD cases vs. controls	2.79 (2.32–3.36)
	YOD cases vs. controls	5.14 (3.70–7.14)
General	UOD cases vs. controls	1.83 (1.79–1.88)
	YOD cases vs. controls	3.33 (2.97–3.74)

*5 categories: 2002–3, 2004–5, 2006–7, 2008–9, 2010–12

This analysis was performed in a 25% random subsample due to computational limitations.
